# Liquid Chromatography Quadrupole Time-of-Flight Mass Spectrometry and Rapid Evaporative Ionization Mass Spectrometry Were Used to Develop a Lamb Authentication Method: A Preliminary Study

**DOI:** 10.3390/foods9121723

**Published:** 2020-11-24

**Authors:** Jishi Wang, Lei Xu, Zhenzhen Xu, Yanyun Wang, Chune Niu, Shuming Yang

**Affiliations:** 1 Key Laboratory of Agro-food Safety and Quality, Institute of Quality Standard & Testing Technology for Agro-Products, Chinese Academy of Agricultural Sciences, Ministry of Agriculture and Rural Affairs, Beijing 100081, China; wjs2018@163.com (J.W.); xulei@cau.edu.cn (L.X.); 82101182143@caas.cn (Y.W.); yangshuming@caas.cn (S.Y.); 2 College of Food Science and Nutritional Engineering, China Agricultural University, Beijing 100083, China; 3 Lanzhou Institute of Husbandry and Pharmaceutical Science, Chinese Academy of Agricultural Sciences, Lanzhou 730050, China; niuchune@caas.cn

**Keywords:** metabolomics, lamb, mutton, UHPLC-QTOF, REIMS

## Abstract

A untargeted metabolomics approach was proposed in this study based on ultra-high performance liquid chromatography quadrupole time-of-flight (UHPLC-QTOF) and rapid evaporative ionization mass spectrometry (REIMS) to discriminate lamb and mutton meat and investigate their subtle metabolic differences, considering the higher popularity of lamb meat than mutton in the market. Multivariate statistical analysis was performed for data processing in order to distinguish between the two sample types. A total of 42 potential metabolites (20 in positive and 22 in negative ion mode) were defined for UHPLC-QTOF analysis, which provided references for discriminating the two kinds of meat. Furthermore, three potential markers were tentatively identified using LC/MS data against chemical databases. In addition, 14 potential metabolites were putatively identified in negative ion mode using the LipidMaps database. Meanwhile, the data-driven soft independent modeling of class analogy (DD-SIMCA) model was established, which could rapidly differentiate non-pretreated lamb meat and mutton with 92% specificity, rendering REIMS a promising technique for meat identification.

## 1. Introduction

Meat is an excellent source of nutrients, such as protein, fat, niacin, and micronutrients [1,2]. However, with the globalization and complication of the food supply chain, meat adulteration has become a severe threat and attracted increasing attention. Adulteration of meat products usually involves the incorporation of or substitution for cheaper ingredients and/or non-compliance with food hygiene regulations in manufacturing, processing, packaging, or storage [3]. In order to seek higher profit, unscrupulous traders often misrepresent meat product information, which infringes upon consumers’ rights [4]. The scandal of substituting horse meat for beef in Europe in 2013 posed a serious consumer trust crisis and attracted global attention as a meat adulteration incident [5]. Researchers conducted a study on 376 Finnish adulteration incidents in 2008 to 2012 notified by Rapid Alert System for Food and Feed and found that most were adulterated animal-derived products [6].

Mutton has become one of the most popular meat products in the global consumer market due to its high-quality protein and low fat and cholesterol contents [7,8]. It is estimated that around 15.123 million tons of mutton were produced worldwide in 2017. Mutton adulterated with low-quality meat has become a public health challenge [9,10]. Lamb meat is derived from sheep at the age of no more than 12 months or without permanent incisor teeth, and mutton is defined as meat from sheep of 1–3 years old [11]. Therefore, lamb meat always has a higher demand and retail price than mutton in the market [12].

So far, many techniques have been developed to detect adulteration and misrepresentation of meat products, such as polymerase chain reaction (PCR) [13], enzyme-linked immunosorbent assay (ELISA) [14], and electronic nose [15]. However, these technologies could not be applied to more subtle or complicated issues, especially adulteration with tissues of the same species. To prevent misrepresenting or mislabeling of mutton products, it is necessary to establish an effective method to distinguish mutton from lamb meat.

Metabolomics, an emerging discipline developed after genomics, transcriptomics, and proteomics, conducts qualitative and quantitative analysis to study the changes in endogenous small molecules caused by disease or external stimuli in biological systems [16]. It has been successfully utilized in the food industry to detect fraudulent activities for the purpose of quality and adulteration verification of meat products [17,18]. Lipidomics, a significant branch of metabolomics, could be employed to detect and quantify the presence of lipids in biological samples [19]. At present, many powerful analytical techniques have been applied to the field of metabolomics, such as gas chromatography-mass spectrometry (GC-MS) [20], liquid chromatography-mass spectrometry (LC-MS) [21], and nuclear magnetic resonance (NMR) spectroscopy [22]. Despite all these approaches, quadrupole time-of-flight mass spectrometry (QTOF) can not only achieve the high-throughput analysis of metabolites but obtain accurate mass numbers of molecular ions and daughter ions, and, therefore, it is highly suitable for the identification of biomarkers in metabolomics research [23].

Rapid evaporative ionization mass spectrometry (REIMS) has recently been developed to achieve semi-quantitative detection of solid samples without any need for sample preparation in a liquid solution and to obtain results immediately by enabling direct ionization from the samples. This technology has been successfully applied to the medical field and food adulteration identification [24,25,26].

In this study, experiments were conducted to compare the metabolic differences in mutton and lamb meat, aiming to find specific markers so as to distinguish them based on ultra-high performance liquid chromatography quadrupole time-of-flight mass spectrometry (UHPLC-QTOF) in parallel with multivariate analysis. In addition, the REIMS technology was utilized to propose a rapid method to distinguish mutton from lamb.

## 2. Materials and Methods

### 2.1. Chemicals and Reagents

Methanol, acetonitrile, butanol, and dichloromethane were purchased from Thermo Fisher Scientific (Thermo Fisher, Waltham, MA, USA), and formic acid and ammonium acetate were from DiKMA Technologies (Beijing, China). Ultrapure water (18.2 MΩ) was prepared using a Milli-Q system (Millipore Billerica, Burlington, MA, USA).

### 2.2. Sample Preparation and Extraction

To verify the feasibility of the method and prevent possible variations in the potential markers in meat samples of different origins, the samples were collected from the same pasture in Sunan County, Gansu Province of China. Eight mutton (3 years old, 50.05 ± 6.96 kg) and eight lamb (8 months, 24.48 ± 2.23 kg) samples were obtained on the day of slaughter. They were fed freely during the grazing period. After removal of longissimus thoracis from each carcass, the samples were transported by a refrigerated van at 4 °C to the laboratory, where they were frozen at −80 °C until use.

The samples for lipidomic analysis were pretreated in accordance with Bligh and Dyer’s method [27] with the following modifications: The longest back muscle samples were separately minced and weighed (~100 mg) into 10 mL centrifuge tubes, and subsequently, 3 mL dichloromethane/methanol (2/1, *v*/*v*) and 2.5 mL ultrapure water were added. The samples were then sonicated for 20 min and centrifuged under 4 °C at 10,000 rpm for 15 min. The subnatant was transferred to a glass centrifuge tube and evaporated to dryness under a gentle nitrogen stream. The samples were re-dissolved in 1 mL methanol and filtered through a syringe filter membrane (0.22 μm) for UHPLC-QTOF analysis.

The quality control (QC) samples were prepared by equal pooling of all pretreated meat samples to monitor the stability and robustness of the measurements, as described in previous studies [28]. During UHPLC-QTOF analysis, the QC samples were inserted and then analyzed after every four real meat samples.

The REIMS technology does not require any form of sample preparation prior to analysis. Its workflow is shown in Figure S1 (Supporting Information).

### 2.3. Untargeted Analysis Based on UHPLC-QTOF

For metabolomic analysis, an ExionLC UHPLC system, which was coupled to 6600 QTOF (Sciex, Redwood, Redwood City, CA, USA), equipped with a C18 column (2.1 × 150 mm, 2.7 μm, Agilent, Santa Clara, CA, USA), was applied. A volume of 5 μL meat extract was injected into the column, the temperature of which was maintained at 40 °C, with the flow rate of 0.3 mL/min. Mobile phase A was water/acetonitrile (15/85, *v*/*v*), and mobile phase B was butanol, both containing 0.1% formic acid and 5 mM ammonium acetate. The gradient was as follows (time, percentage of B): 0 min, 2%; 3 min, 90%; 5 min, 50%; 6 min, 55%; 9 min, 60%; 11 min, 70%; 13 min, 2%; 13–15 min, constantly 2%. Mass spectrometry was implemented using a DuoSpray ion source in positive and negative ionization modes, respectively. Furthermore, QTOF was configured with an automated calibrant delivery system to perform external calibration for every five samples each time using a calibrated solution. The electrospray ionization parameters were optimized: ion source voltage set at 5500 V and −4500 V in positive and negative modes, respectively; the temperature of 550 °C; nebulizer gas (GS 1) of 50 psi; heater gas (GS 2) of 50 psi; declustering potential (DP) of 80 V. The mass spectrometry scan types were classified into TOF MS scan and product ion scan for each sample, which were acquired by the independent-data-acquisition (IDA) mode simultaneously with the mass range of 50–1500. MS/MS analysis was carried out with the collision energy (CE) of 35 V and collision energy spread (CES) of 15 V, meaning 35 ± 15 V.

### 2.4. Experimental Setup of REIMS

A Medimass REIMS source (Medimass, Budapest, Hungary) coupled to Xevo G2-XS Q-TOF mass spectrometer (Waters, Wilmslow, UK), equipped with a monopolar electrosurgical knife (Model PS01-63H, Hangzhou Medstar Technology Co, Ltd., Jiaxing, China), was applied in negative ion and sensitivity modes with continuum data acquisition. The instrument parameter settings referenced Song et al.’s method [29], and MS data were acquired from the range of 50–1200 *m*/*z* with a scan speed of 0.5 s/scan. The generator, Erbe VIO 50C, was operated in auto-cut mode with the power set at 30 W for electrosurgical dissection. Each sample was cut 8–12 times on the return electrode for reproducibility, and each cut lasted approximately 3–5 s. After all data files were pre-processed using a QI-bridge conversion tool, 8–12 independent data were collected for each sample. Prior to operation, the instrument was calibrated for 2 min using 90% isopropanol (IPA) containing 5 mM sodium formate solution, while the flow rate was set at 0.2 mL/min. For accurate mass correction, leucine enkephalin (Leu-Enk) with *m*/*z* 554.2615 (2 ng/µL) in IPA was used as a lock-mass solution, which was injected via a Waters Acquity UPLC I-class system (Waters, Milford, MA, USA) at a rate of 0.1 mL/min. To avoid carryover effects, methanol was employed to clean the iKnife, a venturi device, and a transfer tube for every ten samples analyzed.

### 2.5. Data Processing and Statistical Analysis

Raw data were pre-processed using Peakview 2.2 (AB Sciex, Redwood, CA, USA), involving peak detection and data filtering, conversion, and normalization. The coefficient of variance (CV), expressed in percentage, was calculated for peak areas based on the QC samples. Metabolites with a CV of more than 30% were filtered, and the peak area of each metabolite was used as a variable to carry out a multivariate analysis. The metabolites with missing values > 50% were removed. After the missing values were replaced with half of the minimum positive values in the raw data, all the data were processed by log transformation and Pareto scaling. The *p*-values of the two meat groups were obtained at the same time point for each peak through a *t*-test using SPSS software. After data pre-processing, the data were subjected to multivariate statistical analysis using SIMCA 14.1 software (Umetrics, Umea, Sweden). Principal component analysis (PCA) was performed to identify and exclude outliers, and then orthogonal projection to latent structures discriminant analysis (OPLS-DA) was carried out to separate the samples to the largest extent, synchronously extract the variables with important contributions to the classification, and obtain the variable importance for projection (VIP) values. The metabolites with *p* < 0.05 and VIP > 1 were considered potential biomarkers [30].

For REIMS data analysis, raw data were pre-processed using LIVE ID software (Waters, Budapest, Hungary). Then, a chemometric model was built according to the average spectrum of each sample, which was calculated from the recorded scans for each sample. A lock-mass correction was done with Leu-Enk (*m*/*z* 554.2615) for the resulting data using Progenesis QI software (Waters, Wilmslow, UK). The replicate threshold for total ion count was set to 10,000 for background subtraction. Log transformation and Pareto scaling were also performed before multivariate analysis to achieve normal data distribution and reduce noises. The processed data was exposed to further chemometric functions, such as OPLS-DA, using LIVE ID software. Subsequently, potential characteristic markers were found through matching the LipidMaps database embedded in QI.

In order to extend the application range of the REIMS, the data were converted by QI-bridge and exported to data-driven soft independent modeling of class analogy (DD-SIMCA) to establish a rapid identification model. The DD-SIMCA is a chemometric tool used in Excel and is a pattern for one-class classification [31,32]. DD-SIMCA operated as an Add-In for Microsoft Excel (SIMCA template.xlsb) was employed for the model establishment to identify lamb adulteration based on PCA applied to the training data of a target class.

## 3. Results

### 3.1. PCA for UHPLC-QTOF Data

**Figure 1:**
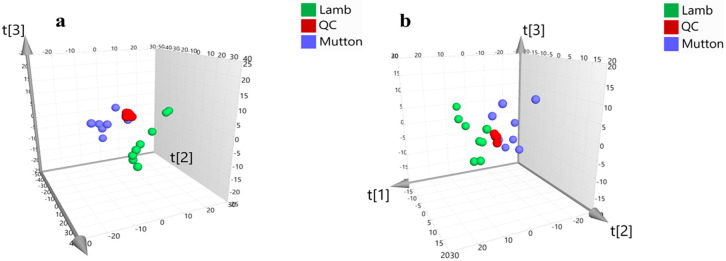
The PCA (principal component analysis) scores plots of compound abundances obtained in positive ion mode (**a**) and negative ion mode (**b**) from samples.

PCA was performed using metabolic data to determine whether the two meat (lamb and mutton) samples could be differentiated. The PCA scores plots (Figure 1) showed that the two groups were separated into independent clusters. The first three principal components (PCs), i.e., PC1, PC2, and PC3, in the age category, explained 29.9%, 13.9%, and 9.2% of the majority variance in positive ion mode and 30.6%, 12.2%, and 10.5% in negative ion mode, respectively. The QC samples were centrally distributed, indicating high stability in the data set. The separation between the two groups was more obvious than the distribution of QC samples, indicating that lamb and mutton were distinguishable.

### 3.2. Differential Abundance Analyses of Two Kinds of Meat Samples

To screen out metabolites with different abundance in the two groups, student *t*-tests and fold changes (FCs) were analyzed. These compounds, with a *p*-value of less than 0.05 and FC value of no less than 2 or no more than 0.5, were considered differently abundant. In this situation, 156 and 200 compounds were identified in positive and negative ion modes, respectively. To further filter and identify potential compounds that contribute significantly to the variation and relevance within the dataset, OPLS-DA (Figure S2 in the Supporting Information) was conducted. The validity of the OPLS-DA model was evaluated through the variation described ability of the model (R^2^(Y)) and the predictive ability of the model (Q^2^(Y)). The models showed a high capability to explain the sample differences (R^2^Y = 0.977 and Q^2^Y = 0.889 in positive ion mode, R^2^Y = 0.974 and Q^2^Y = 0.814 in negative ion mode, respectively). The S-plots constructed from OPLS-DA are shown in Figure S3 (Supporting Information). The variables with an absolute VIP value greater than 1 (points farther from the coordinate origin in S-plots), contributing the most to the classification, were selected as potential markers of interest. In this way, a total of 42 potential metabolites (20 in positive and 22 in negative ion mode) were defined (Table S1 in the Supporting Information).

### 3.3. Tentative Identification of Potential Markers

The ChemSpider database linked to the QI software was used to putatively identify candidate metabolites, and the MS/MS information was utilized to further confirm the structures of potential markers. It was worth mentioning that the number of potential formulas matched could be significantly reduced based on the high resolution and quality accuracy characteristics of TOF-MS [33]. The *m*/*z* and fragmentation pattern simultaneously matched the Human Metabolome Database (HMDB), increasing the likelihood of tentative identification. Ultimately, three potential markers, including flavoxanthin, phosphatidic acid (PA), and phosphatidylinositol (PI), were tentatively annotated, as shown in Table 1.

**Figure 2:**
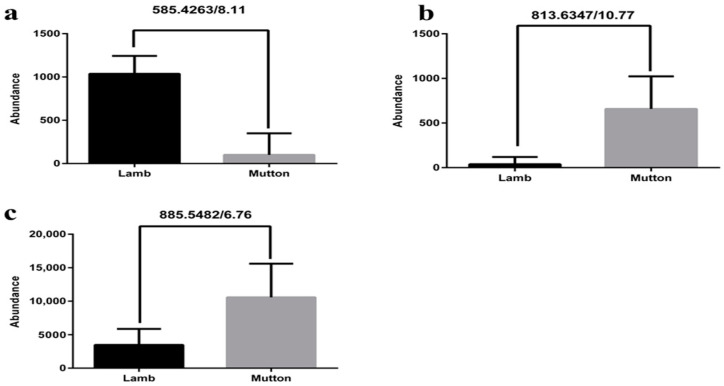
The abundance of potential markers between mutton and lamb. (**a**): Flavoxanthin; (**b**): phosphatidic acid (PA); (**c**): phosphatidylinositol (PI).

Flavoxanthin had a higher abundance in lamb than in mutton (Figure 2a). It is a natural xanthophyll pigment with a golden-yellow color and belongs to the carotenoid containing an oxygenated carotene backbone. It is found in small quantities in a variety of plants, such as dandelion and Medicago falcate (containing 7–8% of flavoxanthin) [34], and is detected in the grazing sheep fat [35]. During the grazing period, flavoxanthin in herbage could be transferred to sheep when it was fed grass on the pasture, such as alfalfa. The difference in the abundance of flavoxanthin in the two kinds of meat may be attributed to the differences in the efficiency of digestion and absorption of grass.

PA had a lower abundance in lamb than in mutton (Figure 2b). PA is a phospholipid (PL) containing -HI, which functions as a common precursor of phospholipids, glycolipids, and storage lipids, participating in extracellular lipid synthesis and playing an important role in lipid synthesis and metabolism. PA is also a signaling molecule widely involved in the regulation of growth, development, and stress.

The results showed that the PI content was lower in lamb than in mutton (Figure 2c), which could be used to distinguish between the two kinds of meat. PI could be obtained from PA under the catalysis of enzymes [36]. In the cell, the 3rd, 4th, and 5th hydroxyl groups on the inositol ring of PI were phosphorylated by the corresponding kinase to form a variety of PI derivatives, which could serve as signal precursors to generate a second messenger [37].

### 3.4. Statistical Analysis for REIMS Data

In this study, REIMS was proposed as a new analytical method for in-situ detection of meat adulteration. For REIMS analysis, both negative and positive ionization modes were considered in the pre-experiment of mutton to detect as many metabolites as possible. During the data collection process, each sample was cut 11 times on the return electrode for reproducibility, and each cut lasted approximately 3–5 s. The classification accuracies of 96% and 90% were observed in the negative and positive ion modes, respectively. To achieve higher accuracy, only the negative ion mode was chosen for REIMS analysis. It was found that clear separation was obtained from the OPLS-DA scores plot between these two groups of meat (Figure S4 in the Supporting Information). The validity of the OPLS-DA model was evaluated through the variation descriptive ability of the model (R^2^(Y)) and the predictive ability of the model (Q^2^(Y)). In the negative ion mode, values obtained for R2(Y) and Q2(Y) were 0.931 and 0.909, respectively, indicating the OPLS-DA model had a good quality. From the S-plots (Figure S5 in the Supporting Information), the ions that were further away from the original contribution (VIP > 1) showed significant separation between the groups, which were regarded as markers.

### 3.5. Potential Markers Identification for REIMS Data

In order to carry out preliminary identification of markers, accurate masses were determined to match the LipidMaps database. Fourteen potential metabolites were putatively identified in negative ion mode. The tentative identification results (Table S2 in the Supporting Information) showed that the most abundant ions were located in the fatty acid and phospholipid regions. In most cases, the same *m*/*z* value provided multiple candidate compounds in the presence of isobaric and isomeric lipid and phospholipid species. Multiple triglyceride (TG), diacylglycerol (DG), and PL achieved separation of lamb and mutton. Age affects the fatty acid composition of meat products. With the growth of age, the body fat deposition increases, fatty acid composition in the fat changes, and then the flavor of mutton changes [38]. In this study, REIMS was explored as a rapid identification technique for mutton and lamb, but limitations were imposed on the acquisition of secondary information of the compounds. Therefore, it was difficult to identify potential markers obtained by REIMS. It should be noted that the technique has the characteristics of immediate data acquisition; hence, this research used data collected by REIMS to establish a discriminative model.

### 3.6. Model Establishment Based on DD-SIMCA

**Figure 3:**
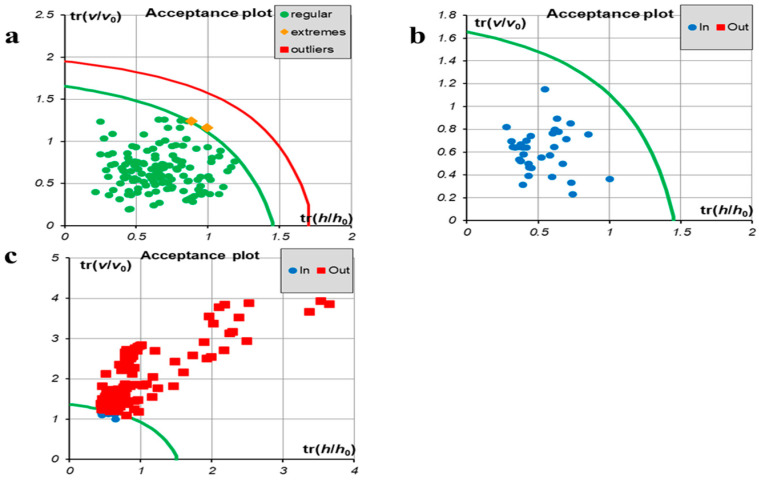
The results of DD-SIMCA (data-driven soft independent modeling of class analogy) classification of meat samples: (**a**) Acceptance plot for the training set, (**b**) Acceptance plot for the test set, (**c**) Acceptance plot for the mutton. Note: where v_0_ and h_0_ are the scaling factors.

DD-SIMCA is an open-source chemometric tool used in Excel and does not require installation. REIMS combined with the DD-SIMCA tool can be used as a rapid identification method for adulteration. During the data collection process, 11 independent data were collected for each sample. Among the processed lamb data, 80% was randomly selected as the training set, which was exported to DD-SIMCA for the model establishment, and the remaining 20% was determined as the test set for evaluating the robustness of the model. Ten PCA components were used to generate a chemometric model. The parameter α (0 < α < 1) was applied to manage the size of the acceptance area. To be specific, a less α value indicates a wider area and more accepted samples. γ (0 < γ < 1) is the value of the outlier significance employed to control the size of the outlier area. The less γ is, the wider the area is, and the fewer the discovered outliers are. In this model, the values of α and γ are 0.02 and 0.05, respectively. The detailed information of the acceptance area could be found previously [39]. Figure 3a shows the acceptance plot for the training set with a sensitivity of 99%, indicating that all training objects were located inside the acceptance area as expected. The plot demonstrated all training samples together with the borders of the acceptance (green) and outlier (red) areas. The samples were colored with respect to their status: regular objects (green dots) belonged to the acceptance area, outliers (red squares) were outside the outlier areas, and extreme samples (yellow diamonds) were located in between. The acceptance plot (Figure 3b) showed all test samples together with the borders of the acceptance (green) area, revealing that the model was robust. The mutton data were then imported into the model, and the results showed that all mutton samples were out of the acceptance area (red squares shown in Figure 3c), implying the specificity of 92%. What that means is the lamb samples were completely separated from the mutton ones, without any overlapping between the two classes. Therefore, the sensitivity and specificity of the model were satisfactory.

## 4. Conclusions

A high-throughput metabolomic approach based on UHPLC-QTOF in combination with multivariate data analysis provided a possible way for distinguishing lamb from mutton, and REIMS, featuring no sample preparation and near-instantaneous results, was also successfully tested in this study. The two kinds of meat could be separated from the PCA scores plot drawn based on LC/MS data. Three potential markers were filtered from the OPLS-DA model and tentatively annotated by matching the databases, and the differences in the REIMS result mainly laid in the fatty acid and phospholipid regions. These different metabolites provided references for the identification of lamb meat and mutton. Moreover, due to the lack of detection standards, further work is required to validate these markers in the following study. Last but not least, in addition to being affected by age, the samples were also related to factors, such as gender and feeding methods. Therefore, adding commercial samples collected from some representative geographic regions to the model is recommended for subsequent study. It is also important that this research highlights the potential of these two technologies to identify markers to distinguish between the two kinds of meat.

## Figures and Tables

**Table 1:** The discriminated potential markers between lamb and mutton.

Compound	Formula	Accurate Mass (Da)	RT (min)	Mode	Fold Change (Lamb/Mutton)	VIP-Value
Flavoxanthin	C40H56O3	585.4263	8.11	Positive	10.36	2.85
Phosphatidic acid (22:2(13Z,16Z)/22:0)	C47H89O8P	813.6347	10.77	Positive	0.06	2.34
Phosphatidylinositol (18:0/20:4(5Z,8Z,11Z,14Z))	C47H83O13P	885.5482	6.76	Negative	0.33	1.26

## Supplementary Materials

The following are available online at https://www.mdpi.com/2304-8158/9/12/1723/s1, Figure S1: The workflow of REIMS analysis; Figure S2: The OPLS-DA scores plots of compound abundances obtained in positive ion mode (a) and negative ion mode (b) for UPLC-QTOF date; Figure S3: S-plots of differential compound abundances obtained in positive (a) and negative ion (b) modes from UHPLC-QTOF data (Red triangle represents VIP > 1); Figure S4: The OPLS-DA scores plots of compound abundances obtained in negative ion modes for REIMS date from lamb and mutton; Figure S5: S-plots of differential compound abundances obtained in negative ion modes from REIMS data from lamb and mutton (Red triangle represents VIP > 1); Table S1: The tentatively discriminated potential markers between lamb and mutton in positive ion mode (a) and negative ion mode (b); Table S2: The tentatively discriminated potential markers between lamb and mutton for REIMS date.
