# Supplementary material for: Liquid Chromatography Quadrupole Time-of-Flight Mass Spectrometry and Rapid Evaporative Ionization Mass Spectrometry Were Used to Develop a Lamb Authentication Method: A Preliminary Study
Source: Foods. 2020 Nov 24;9(12):1723. doi: 10.3390/foods9121723 (PMC7761048; doi:10.3390/foods9121723)
Supplement: Supplementary file 1 [file foods-09-01723-s001.pdf]

Supplymentary material

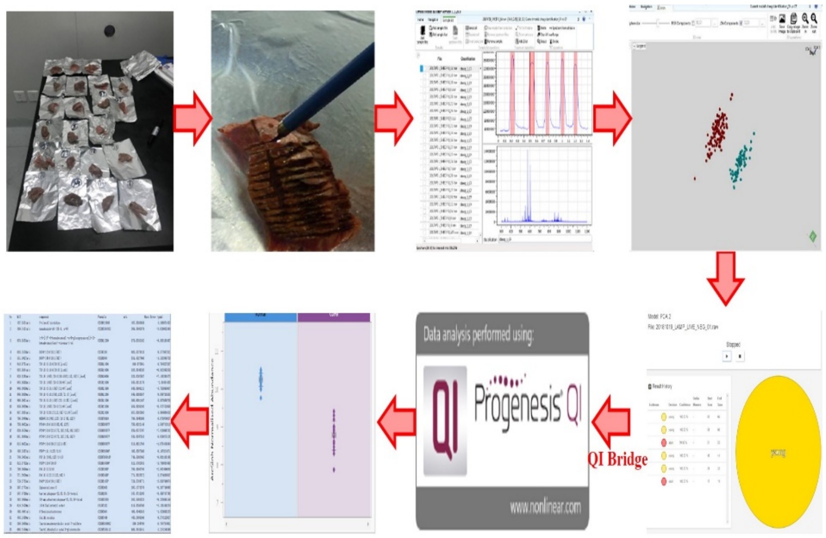

Figure S1. The workflow of REIMS analysis.

(a)

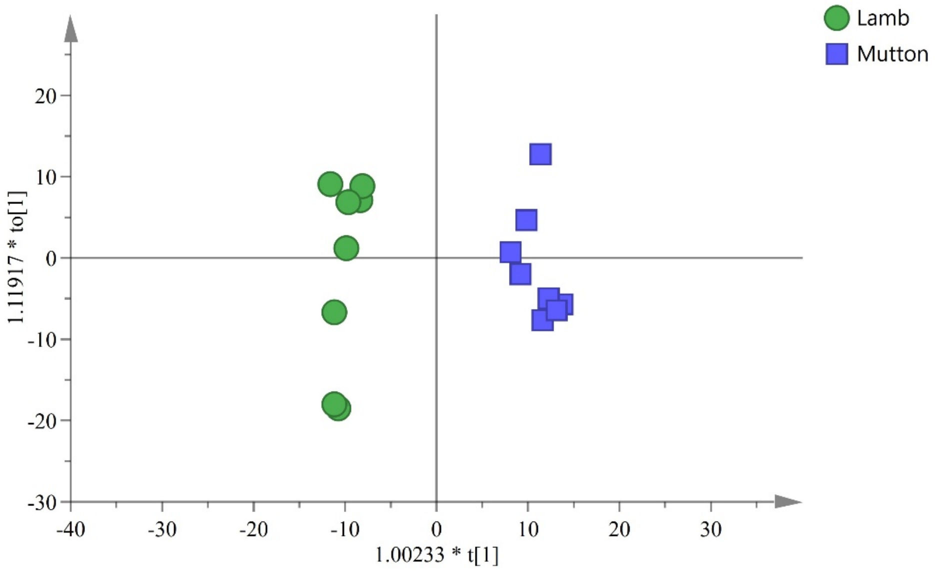

(b)

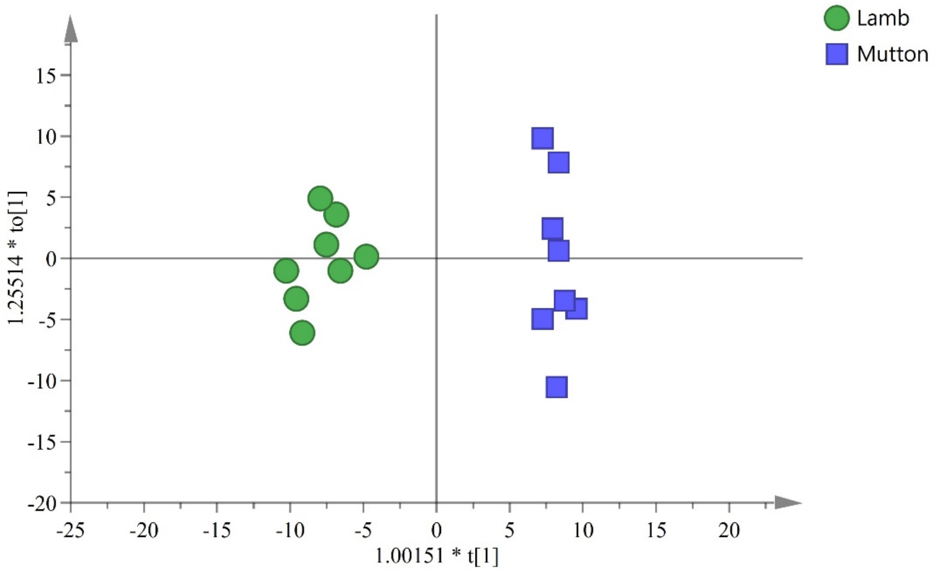

Figure S2. The OPLS-DA scores plots of compound abundances obtained in positive ion mode (a) and negative ion mode (b) for UPLC-QTOF date.

(a)

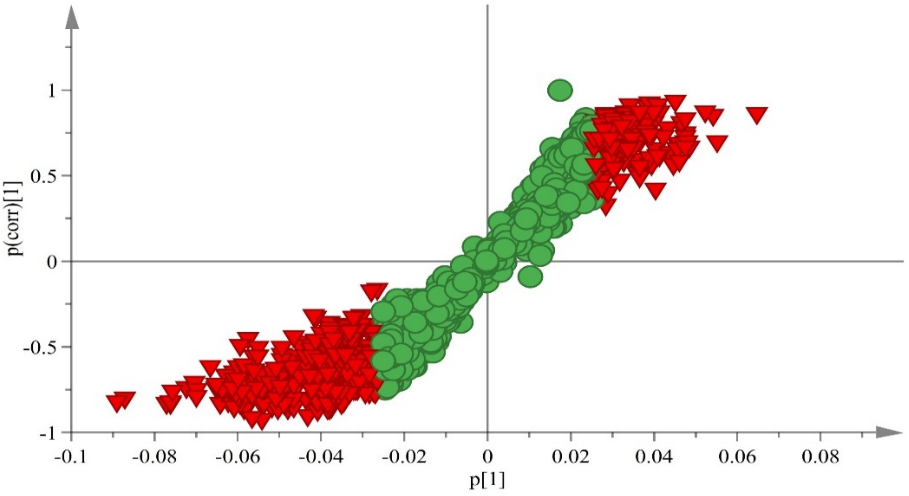

(b)

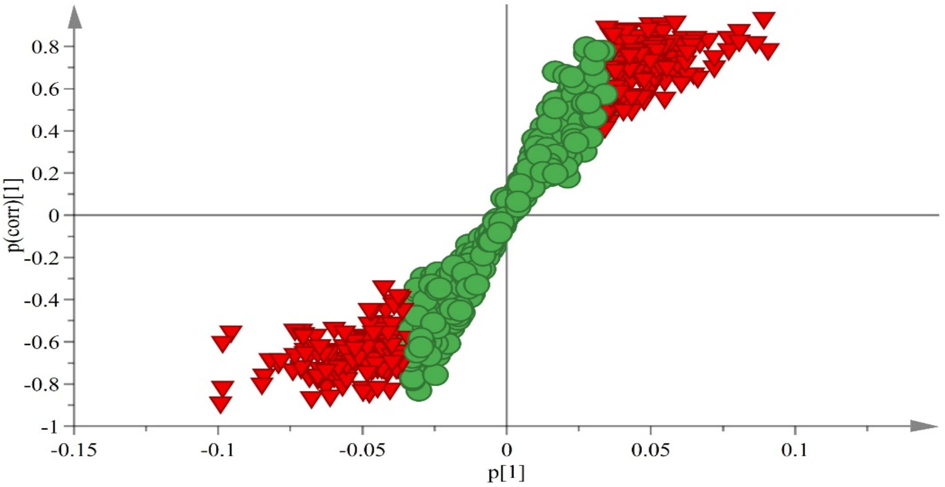

Figure S3. S-plots of differential compound abundances obtained in positive (a) and negative ion (b) modes from UHPLC-QTOF data (Red triangle represents VIP>1).

Table S1. The tentatively discriminated potential markers between lamb and mutton in positive ion mode (a) and negative ion mode (b).

(a)

	Accurate mass (Da)	RT (min)	Higher Abundance	Lower Abundance	VIP-value
1	357.2487	1.34	Mutton	Lamb	1.15
2	179.1292	1.4	Mutton	Lamb	2.05
3	673.3396	1.63	Lamb	Mutton	3.43
4	717.3685	1.63	Lamb	Mutton	3.51
5	849.4439	1.63	Lamb	Mutton	1.26
6	189.1335	1.69	Mutton	Lamb	1.87
7	478.1444	1.75	Mutton	Lamb	1.39
8	295.0883	3.56	Mutton	Lamb	1.02
9	260.1701	4.48	Lamb	Mutton	2.23
10	582.2932	4.69	Lamb	Mutton	1.55
11	72.0803	5.13	Lamb	Mutton	1.34
12	295.0872	6.17	Lamb	Mutton	1.48
13	416.3355	7	Mutton	Lamb	1.79
14	442.351	7.08	Mutton	Lamb	1.63
15	391.25	7.57	Lamb	Mutton	1.95
16	529.4598	8.11	Lamb	Mutton	2.98
17	569.4525	8.11	Lamb	Mutton	2.58
18	585.4263	8.11	Lamb	Mutton	2.85
19	813.6347	10.77	Mutton	Lamb	2.34
20	823.6759	10.81	Lamb	Mutton	1.62

(b)

	Accurate mass (Da)	RT (min)	Higher Abundance	Lower Abundance	VIP-value
1	212.0228	1.22	Mutton	Lamb	1.62
2	347.038	3.11	Mutton	Lamb	1.36
3	664.1161	3.44	Lamb	Mutton	2.47
4	244.1368	3.73	Lamb	Mutton	2.91
5	386.9368	5.65	Mutton	Lamb	2.04
6	312.8826	5.67	Mutton	Lamb	1.46
7	346.8984	5.68	Mutton	Lamb	1.65
8	398.8671	5.68	Mutton	Lamb	1.92
9	500.9156	5.69	Mutton	Lamb	2.65

10	244.9092	5.72	Mutton	Lamb	1.29
11	272.9581	5.72	Mutton	Lamb	1.61
12	278.9238	5.72	Mutton	Lamb	1.35
13	270.9778	5.86	Mutton	Lamb	1.88
14	272.958	6.26	Mutton	Lamb	1.27
15	286.9727	6.28	Mutton	Lamb	1.42
16	885.5482	6.76	Mutton	Lamb	1.26
17	511.2211	6.86	Mutton	Lamb	1.31
18	861.548	6.86	Mutton	Lamb	1.04
19	863.5634	7.09	Mutton	Lamb	1.26
20	625.3254	7.8	Lamb	Mutton	1.84
21	783.4383	9.36	Lamb	Mutton	2.30
22	487.2007	14.69	Lamb	Mutton	1.41

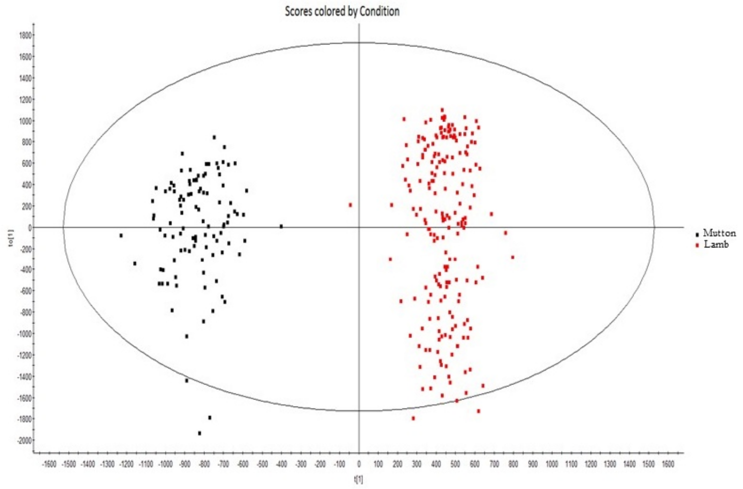

Figure S4. The OPLS-DA scores plots of compound abundances obtained in negative ion modes for REIMS date from lamb and mutton.

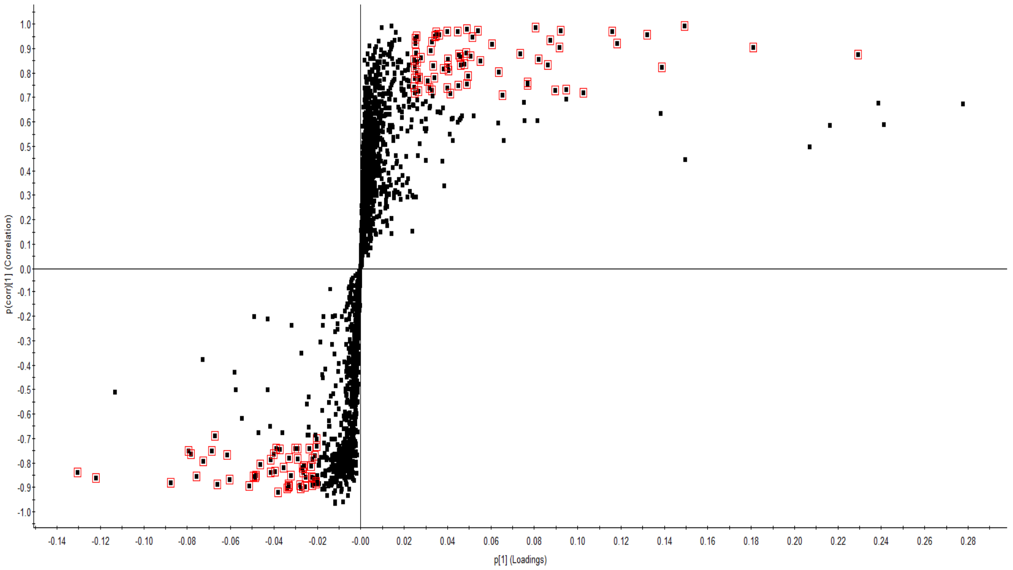

Figure S5. S-plots of differential compound abundances obtained in negative ion modes from REIMS data from lamb and mutton (Red triangle represents VIP>1).

Table S2. The tentatively discriminated potential markers between lamb and mutton for REIMS date.

	m/z	Formula	Adducts	Description
1	952.77	C56H108NO8P	M-H	PC(24:1(15Z)/24:1(15Z))
2	1003.73	C69H98O6	M-H2O-H	TG(22:6(4Z,7Z,10Z,13Z,16Z,19Z)
3	533.46	C34H64O5	M-H2O-H	DG(15:0/16:1(9Z)/0:0)
4	563.50	C36H70O5	M-H2O-H	DG(15:0/18:0/0:0)
5	653.45	C37H69O8P	M-H2O-H	PA(16:0/18:2(9Z,12Z))
6	648.63	C42H83NO3	M-H	Ceramide (d18:1/24:0)
7	1005.75	C69H100O6	M-H2O-H	TG(22:6(4Z,7Z,10Z,13Z,16Z,19Z)
8	535.47	C34H66O5	M-H2O-H	DG(15:0/16:0/0:0)
9	331.26	C22H36O2	M-H	Adrenic acid
10	561.49	C36H68O5	M-H2O-H, M-H	DG(15:0/18:1(11Z)/0:0)
11	559.47	C36H66O5	M-H2O-H	DG(15:0/18:2(9Z,12Z)/0:0)
12	587.50	C38H70O5	M-H2O-H	DG(16:0/0:0/18:2n6)
13	601.46	C38H66O5	M-H2O-H, M-H	DG(15:0/20:4(5Z,8Z,11Z,14Z)/0:0)
14	585.48	C38H68O5	M-H2O-H	DG(15:0/20:3(5Z,8Z,11Z)/0:0)
